# An Underwater Image Enhancement Method for Different Illumination Conditions Based on Color Tone Correction and Fusion-Based Descattering

**DOI:** 10.3390/s19245567

**Published:** 2019-12-16

**Authors:** Yidan Liu, Huiping Xu, Dinghui Shang, Chen Li, Xiangqian Quan

**Affiliations:** 1 School of Ocean and Earth Science, Tongji University, Shanghai 200092, China; 1510853@tongji.edu.cn (Y.L.); 1410845@tongji.edu.cn (D.S.); 2 Institute of Deep-Sea Science and Engineering, Chinese Academy of Sciences, Hainan 572000, China; lic@idsse.ac.cn (C.L.); quanxq@idsse.ac.cn (X.Q.)

**Keywords:** underwater image enhancement, color correction, contrast enhancement, underwater artificial illumination, deep-sea image

## Abstract

In the shallow-water environment, underwater images often present problems like color deviation and low contrast due to light absorption and scattering in the water body, but for deep-sea images, additional problems like uneven brightness and regional color shift can also exist, due to the use of chromatic and inhomogeneous artificial lighting devices. Since the latter situation is rarely studied in the field of underwater image enhancement, we propose a new model to include it in the analysis of underwater image degradation. Based on the theoretical study of the new model, a comprehensive method for enhancing underwater images under different illumination conditions is proposed in this paper. The proposed method is composed of two modules: color-tone correction and fusion-based descattering. In the first module, the regional or full-extent color deviation caused by different types of incident light is corrected via frequency-based color-tone estimation. And in the second module, the residual low contrast and pixel-wise color shift problems are handled by combining the descattering results under the assumption of different states of the image. The proposed method is experimented on laboratory and open-water images of different depths and illumination states. Qualitative and quantitative evaluation results demonstrate that the proposed method outperforms many other methods in enhancing the quality of different types of underwater images, and is especially effective in improving the color accuracy and information content in badly-illuminated regions of underwater images with non-uniform illumination, such as deep-sea images.

## 1. Introduction

**Figure 1:**
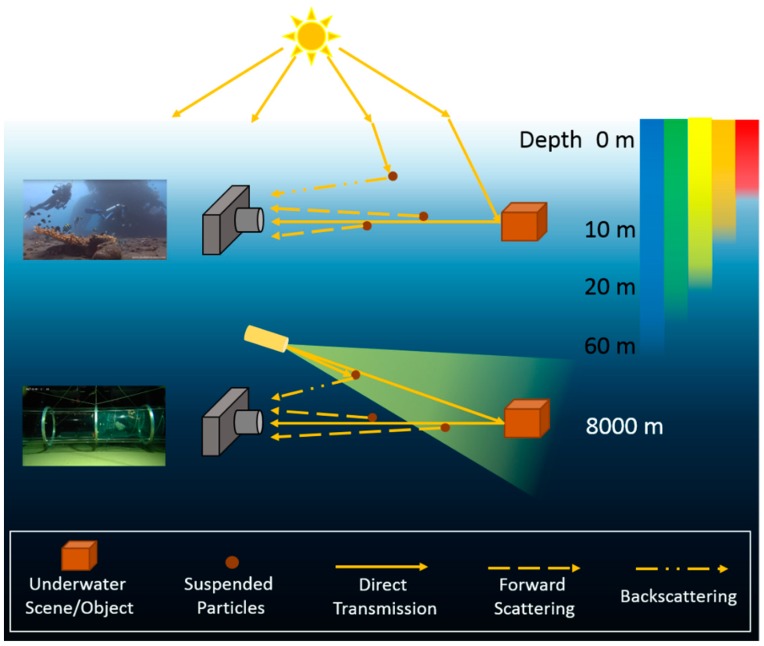
Underwater optical imaging in shallow water and deep sea.

As one of the most direct approaches to perceive the world below the surface, optical images can provide plenty of useful information for various underwater applications, such as marine geology surveys, underwater mining, fishery and marine archaeology [1,2,3,4,5]. However, compared with images from terrestrial environment, underwater images often suffer from severe degradation problems, which can impact their reliability and utility in underwater applications. The reason of underwater image degradation is in many aspects. The first one is the degradation caused by the water body. As shown in Figure 1, light with different wavelengths is attenuated in different ratios in the water body. This uneven attenuation leads to ubiquitous color bias in underwater images. Suspended particles in water can also cause degradation in underwater images. As depicted in Figure 1, particles near the transmission path from photographed scene to camera cause small-angle scattering (forward scattering) of incident light, and particles in surrounding environment induce ambient light into the camera lens by large-angle scattering (backscattering). These redirections of light lead to blurriness and hazy looks in underwater images. As the going deep of underwater explorations, artificial lighting devices are added to provide necessary illumination for dark deep-sea environment, as depicted in Figure 1. Due to the limited range and inhomogeneity of artificial illumination, problems like dark background and bright spot are often seen in deep-sea images. Besides, if the light source is chromatic, the color balance of underwater images will also be affected. To facilitate the usage of underwater images, these degradation problems should be addressed.

To date, there have been many attempts to improve the quality of underwater images. According to recent literature reviews [6,7], they are usually classified into two main categories: underwater image enhancement and underwater image restoration. Methods of underwater image enhancement require no prior knowledge about the environment and mainly aim at improving the visual quality of underwater images. For example, in [8], Iqbal et al. used histogram stretching in the RGB color space to restore the color balance, and the stretching of saturation and intensity in the HSI color space to improve the colorfulness and contrast of underwater images. In [9], Ancuti et al. proposed a multi-scale fusion method that combined the result images of color correction and contrast enhancement by four weight maps about image luminance, contrast, chromatic and saliency. In [10], Ghani and Isa reduced the color deviation in underwater images by using the characteristics of Rayleigh distribution, and improved the image saturation and contrast by stretching corresponding components in the HSV color space. In [11], Li et al. proposed a weakly supervised color transfer method to correct color distortion in underwater images. 

Underwater image restoration methods, on the other hand, attempt to recover the true scene radiances from degraded underwater images. These methods use models to analyze the mechanism of underwater image degradation, and restore the images by reversing the degradation process and using model parameters deduced via prior knowledges. In these methods, the simplified image formation model (IFM) is frequently used for its effectiveness and simplicity [6], and due to its similarity to the model of outdoor hazy image, the Dark Channel Prior (DCP) from outdoor image dehazing [12] is also widely introduced in methods based on this model. In [13], Galdran et al. proposed the Red Channel Prior based on the DCP to recover the lost contrast in underwater images. This new prior reversed the red channel to deal with the strong attenuation of red light in the water body. In [14], Drews Jr et al. proposed the Underwater DCP (UDCP) from the traditional DCP by excluding the red channel in producing the prior. Apart from the DCP-related priors, there are also other priors proposed for underwater image restoration. In [15], Carlevaris-Bianco et al. proposed a prior by comparing the maximum intensity of the red channel to the maximum intensity in the green and blue channels over a small image patch. In [16] and [17], Peng et al. defined a new prior from image blurriness and used it to improve the quality of images from various underwater environments. There are also some other methods that combined the features of former two categories. For example, in the work of Hou et al. [18], the UDCP is used together with quad-tree subdivision and Gamma correction to improve the contrast and saturation of underwater images. And in [19], Qing et al. proposed a comprehensive method with adaptive dehazing and adaptive histogram equalization to remove the scattering and restore the color balance of underwater images. Usually for these methods, no strict classification is made.

From the study of the relevant literature, we have also noticed that most of the published works are designed for solving water-caused problems, i.e., color deviation and low contrast caused by the attenuation and scattering of light in the water body, while only a few of them have considered the degradation caused by artificial lighting. Moreover, in latter works, the study of lighting-caused degradation is also seemed to be limited in the range of local problems like bright spots [13] or vignetting [20], but more general problems like the influence to the distribution of color and brightness in the whole image range are rarely studied.

In this paper, we propose an underwater image enhancement method for different illumination conditions based on a new model of underwater image degradation. In the new model, illumination is included in the modeling of the single-pixel intensity, so its influences to local regions as well as the whole image range are covered in this model. The proposed method is composed of two components: color-tone correction and fusion-based descattering. The first component is based on a frequency-based color-tone estimation strategy. By changing its application range and using necessary modification filters, it can be used to correct the global color cast in uniformly-illuminated images and regional color cast in non-uniformly-illuminated images. The second component is used to solve the residual degradation problems that are related to the scene-camera distance. This component adopts a fusion strategy to enhance images under different states. Experiments on laboratory and open-water images of different depths and lighting conditions prove the effectiveness of the proposed method. According to qualitative and quantitative evaluation results, the proposed method can improve the color balance and contrast of underwater images, and restore the color accuracy and visibility of badly-illuminated regions in non-uniformly illuminated images.

The rest of this paper is organized as follows: In Section 2, the enhancement of underwater images with different illumination conditions is studied theoretically based on the model study of underwater images with arbitrary illumination conditions. In Section 3, the overall framework and individual components of the proposed method are introduced. In Section 4, the proposed method is evaluated on images from shallow water, laboratory and deep sea images, with comparison to three state-of-the-art underwater image enhancement and restoration methods. And in the last section, conclusions of this work are presented.

## 2. Problem Formulation and Model Improvement

To study the solution of underwater image degradation problems caused by the water body and the light source, the Jaffe–McGlamery model [21,22], a general model of underwater image formation, is reviewed first. In the Jaffe–McGlamery model, the irradiance of a monochromatic underwater image is formulated as the linear combination of following three components: the direct component $E_{d}$, the forward scattering component $E_{fs}$ and the backscattering component $E_{bs}$, i.e.,

(1) $$E\left(x|\lambda \right)=E_{d}\left(x|\lambda \right)+E_{fs}\left(x|\lambda \right)+E_{bs}\left(x|\lambda \right),$$

where x represents an image point, and λ is the wavelength of incident light. By weighing with the spectral response [23] of the detector, the monochromatic irradiances of the whole spectrum are integrated and transformed into the pixel values of an image in the RGB color space, i.e., $I^{k}\left(x\right)=\int_{\lambda }Q^{k}\left(\lambda \right)\phi^{k}\left(\lambda \right)E\left(x|\lambda \right)d\lambda$, where $\phi^{k}\left(\lambda \right)$ is the spectral response of channel k, k∈{R,G,B}, and $Q^{k}\left(\lambda \right)$ is a factor about the imaging system and the unit conversion from light irradiance to pixel intensity.

In the model of monochromatic image irradiance (i.e., Equation (1)), the direct component $E_{d}\left(x|\lambda \right)$ corresponds to the irradiance that has been exponentially decayed after being reflected by the photographed scene, i.e.,

(2) $$E_{d}\left(x|\lambda \right)=E_{I}\left(x|\lambda \right)M(x|\lambda )e^{-c(\lambda )d\left(x\right)}\kappa \left(x\right),$$

where $E_{I}\left(x|\lambda \right)$ is the irradiance of incident light on the scene point, M(x|λ) is the reflectance of the scene point, c(λ) is the volume attenuation coefficient, d(x) is the distance between the scene point and the camera, and κ(x) represents other parameters of the imaging system. According to [22], $E_{I}\left(x|\lambda \right)$ is a function of the light source irradiance and the attenuation along the transmission path from the light source to the scene. Using BP to represent the beam pattern of the light source, γ to represent the angle from the light source to the scene point, and D to represent the distance between the scene point and the light source, the incident irradiance $E_{I}\left(x|\lambda \right)$ is calculated as $E_{I}(x|\lambda )=BP\left(x|\lambda \right)cos\gamma \left(x\right)\;e^{-c(\lambda )D\left(x\right)}/D^{2}\left(x\right)$. The formula of κ(x) is given by $\kappa \left(x\right)=\cos^{4}\vartheta \left(x\right)\cdot \frac{T_{l}}{4f_{n}^{2}}\cdot {\left[\frac{d\left(x\right)-F_{l}}{d\left(x\right)}\right]}^{2}$, where ϑ is the angle from the camera to the scene point, $T_{l}$ is the transmittance of the lens, and $f_{n}$ is the F number of the camera of focal length $F_{l}$. 

The forward scattering component $E_{fs}\left(x|\lambda \right)$ corresponds to the reflected light with small-angle scattering. It is calculated by convoluting the direct component with a point spread function g(x|λ):

(3) $$E_{fs}\left(x|\lambda \right)=E_{d}\left(x|\lambda \right)*g\left(x|\lambda \right).$$

Here, “∗” denotes convolution, $g\left(x|\lambda \right)=\left[e^{-G\left(\lambda \right)d\left(x\right)}-e^{-c\left(\lambda \right)d\left(x\right)}\right]F^{-1}\left\{e^{-ℬ\left(\lambda \right)d\left(x\right)f}\right\}$, where G and ℬ are empirical constants, $F^{-1}\left\{\cdot \right\}$ represents the inverse Fourier transform, and f is radial frequency. According to [22], a more accurate representation of $E_{I}$ in Equation (2) should also include the forward scattering process, i.e., ${E_{I}}^{\prime }\left(x|\lambda \right)=E_{I}\left(x|\lambda \right)\;+E_{I}\left(x|\lambda \right)*g\left(x|\lambda \right),$ where ${E_{I}}^{\prime }$ represents the more accurate representation of $E_{I}$.

The final component, backscattering $E_{bs}\left(x|\lambda \right)$, corresponds to the light that enters the camera without being reflected by the scene. In its original form, it is calculated as the superposition of the illuminated volume elements weighted by the value of the volume-scattering function [22]:

(4) $$E_{bs}\left(x|\lambda \right)=\overset{N}{\underset{i=1}{\sum }}e^{-c(\lambda )Z_{i}}\beta \left(\phi \right)E_{s}\left(x|\lambda \right)\kappa^{\prime }\left(x\right),$$

where β(ϕ) is the volume-scattering function, $E_{s}\left(x|\lambda \right)$ is the irradiance received by the small volume element in layer i, $Z_{i}$ is the distance from the layer i to the camera, and $\kappa^{\prime }\left(x\right)$ represents parameters of the camera system. According to [22], $E_{s}(x|\lambda )$, like $E_{I}(x|\lambda )$ of Equation (2), is a function of the source light and the attenuation along the source-to-scene path; mathematically, $E_{s}(x|\lambda )=BP\left(x|\lambda \right)\;e^{-c(\lambda )D_{i}\left(x\right)}/D_{i}^{2}\left(x\right)$, where $D_{i}$ is the distance from the element in layer i to the light source. Forward scattering should also be included for a more accurate calculation of $E_{s}$. $\kappa^{\prime }\left(x\right)$ is similar to κ(x) in Equation (2) and is calculated by $\kappa^{\prime }\left(x\right)=\cos^{3}\vartheta_{i}\left(x\right)\cdot \frac{\pi \Delta ZT_{l}}{4f_{n}^{2}}\cdot {\left[\frac{Z_{i}-F_{l}}{Z_{i}}\right]}^{2}$, where $\vartheta_{i}$ is the angle from the camera to the element in layer i, and ΔZ is the sickness of the layer.

In [24], a simplified formula for calculating backscattering in uniformly illuminated conditions is provided, which is

(5) $$E_{bs}\left(x|\lambda \right)=E_{bs,\infty }\left(\lambda \right)\left[1-e^{-c(\lambda )d\left(x\right)}\right],$$

where $E_{bs,\infty }$ represents the backscattering of infinite distance. According to [24], it is a function of incident irradiance, the reciprocal of volume attenuation coefficient and the full-angle integral of volume scattering function, i.e., $E_{bs,\infty }\left(\lambda \right)\propto \frac{E_{s}\left(\lambda \right)}{c\left(\lambda \right)}\int_{\Theta }\beta \left(\phi \right)d\phi$.

Clearly, in the Jaffe–McGlamery model, water-caused degradation and the influence of light source are well covered, which makes it a good simulation tool for general underwater images. But for the task of underwater image restoration, its complexity hinders its usage. Instead, a simplified version, the IFM model, is more commonly used to restore underwater images, as mentioned in the former section. The formula of the IFM model is given as follows:

(6) $$I^{k}\left(x\right)=J^{k}\left(x\right)t^{k}\left(x\right)+B^{k}\left[1-t^{k}\left(x\right)\right],\;k\in \left\{R,G,B\right\}$$

where $I^{k}\left(x\right)$ is the underwater image, $J^{k}\left(x\right)$ is the target image that represents scene radiance, $t^{k}\left(x\right)$ is the transmission map (TM) that equals to $e^{-c^{k}d\left(x\right)}$, and $B^{k}$ is the background light (BL). For IFM-based methods, the restoration of an underwater image is achieved by estimating and eliminating TM and BL via proper priors about the input underwater image. 

By comparing the Jaffe–McGlamery model and the IFM model, it’s very clear that the IFM model is an approximation of the Jaffe–McGlamery model in the case of uniform illumination. In the IFM model, the product of $E_{I}\left(x|\lambda \right)$ and M(x|λ) is replaced by $J^{k}\left(x\right)$, and the original form of the backscattering component is replaced by the simplified formula in Equation (5). The forward scattering component and the other parameters are omitted because of their relatively low influence on the value of pixel intensity, and the integral of irradiances of the whole spectrum is simplified by using corresponding parameters of each color channel (i.e., $c^{k}$ and $B^{k}$) to keep the solvability of the model. Apparently, the IFM model can be invalid in the condition of inhomogeneous illumination, because the condition of using Equation (5) is not satisfied. Moreover, the estimated scene radiance can also be inaccurate due to the discrepancy of $E_{I}$ from normal daylight. To improve the model accuracy while remaining its simplicity, we make a small change to the IFM model by adding a parameter to represent incident light. The modified model is given as follows:

(7) $$I^{k}\left(x\right)=L^{k}\left(x\right)J^{k}\left(x\right)t^{k}\left(x\right)+b^{k}\left(x\right),\;k\in \left\{R,G,B\right\}.$$

Here $L^{k}\left(x\right)$ stands for the incident light of the scene that corresponds to $E_{I}\left(x|\lambda \right)$ for $E_{d}\left(x|\lambda \right)$. $b^{k}\left(x\right)$ corresponds to the $E_{bs}\left(x|\lambda \right)$ in Equation (4). In the case of uniform illumination, it is simplified as $B^{k}\left[1-t^{k}\left(x\right)\right]$, as in the IFM model.

For the case of inhomogeneous illumination, the simplification of backscattering cannot be directly applied, but based on the assumption that light field changes gradually in space, the inhomogeneous illumination can be approximated as homogeneous in small regions. By applying the simplification of backscattering to these small regions, the above model is transformed into:

(8) $$I^{k}\left(x\right)=L^{k}\left(\Omega_{x}\right)J^{k}\left(x\right)t^{k}\left(x\right)+B^{k}\left(\Omega_{x}\right)\left[1-t^{k}\left(x\right)\right],\;k\in \left\{R,G,B\right\},$$

where $\Omega_{x}$ represents a small neighborhood of x that receives homogeneous illumination. Apparently, the case of homogenous illumination that is assumed in the IFM model is a special case of this new model.

Due to the uncertainty and spatial variance of $L^{k}\left(\Omega_{x}\right)$ and $B^{k}\left(\Omega_{x}\right)$, priors for solving $J^{k}\left(x\right)$ from the new model are hard to derive. However, since $L^{k}\left(\Omega_{x}\right)$ and $B^{k}\left(\Omega_{x}\right)$ mainly cause color deviation in the regional or full-image scale, their influence can be suppressed by the idea of white balance under the Gray World assumption. For an image with uniform illumination and invariant scene-camera distance, i.e., $I^{k}\left(x\right)=L^{k}J^{k}\left(x\right)t^{k}+B^{k}\left(1-t^{k}\right)$, white balance can be easily applied with a linear transformation whose general form is $I_{T}^{k}\left(x\right)=\left[I^{k}\left(x\right)-H_{1}^{k}\right]/H_{2}^{k}$, because all the terms that result in color deviation in $I^{k}\left(x\right)$ (i.e., $L^{k}$, $t^{k}$ and $\;B^{k}$) are global constants in this model. In the general formula of linear transformation, $H_{1}^{k}$ and $H_{2}^{k}$ are abstract parameters that respectively represent the general effects of offsetting and scaling processes. These abstract parameters need to be concretized according to the color-deviation characters of $I^{k}\left(x\right)$ to ensure an unbiased transformation result. For example, in the color-tone correction method presented in the next section, the offsetting ($H_{1}^{k})$ and scaling ($H_{2}^{k})$ processes are performed with a subtraction of the biased color tone and a classic histogram stretching, and the subtracted color-tone image is estimated on the basis of the frequency character of $I^{k}\left(x\right)$ to approximate the true color-deviation condition of $I^{k}\left(x\right)$. 

For an image with uniform illumination and variant scene-camera distance, i.e., $I^{k}\left(x\right)=L^{k}J^{k}\left(x\right)t^{k}\left(x\right)+B^{k}\left[1-t^{k}\left(x\right)\right]$, a similar linear transformation can remove the color deviation resulted from $L^{k}$ and $B^{k}$, and partly from $t^{k}\left(x\right)$, because former two terms are spatial constants and the last term is not. For the case of non-uniform illumination, i.e., $I^{k}\left(x\right)=L^{k}\left(\Omega_{x}\right)J^{k}\left(x\right)t^{k}\left(x\right)+B^{k}\left(\Omega_{x}\right)\left[1-t^{k}\left(x\right)\right]$, the color cast caused by $L^{k}\left(\Omega_{x}\right)$ and $B^{k}\left(\Omega_{x}\right)$ can still be suppressed by linear transformation in region $\Omega_{x}$, based on the assumption that the illumination is uniform in $\Omega_{x}$. But in practice, the criterion of uniform illumination cannot be applied very strictly, because the Gray World assumption can be invalid in extremely small regions with colorful objects. So instead of suppressing the regional constants $L^{k}\left(\Omega_{x}\right)$ and $B^{k}\left(\Omega_{x}\right)$, it is more practical to apply linear transformation by pixels, with corresponding parameters estimated from loosely-defined uniform regions and refined by smoothing filters to compensate the inter-regional differences.

After linear transformation, the global or regional color-tone deviation related to incident light is suppressed. To solve the residual problems about $t^{k}\left(x\right)$, the result image is further transformed into the following form:

(9) $$\begin{array}{ll} I_{T}^{k}\left(x\right) & =\frac{L^{k}\left(\Omega_{x}\right)J^{k}\left(x\right)-H_{1}^{k}\left(x\right)}{H_{2}^{k}\left(x\right)}t^{k}\left(x\right)+\frac{B^{k}\left(\Omega_{x}\right)-H_{1}^{k}\left(x\right)}{H_{2}^{k}\left(x\right)}\left[1-t^{k}\left(x\right)\right]\\ & =J_{T}^{k}\left(x\right)t^{k}\left(x\right)+B_{T}^{k}\left(x\right)\left[1-t^{k}\left(x\right)\right],\;k\in \left\{R,G,B\right\}. \end{array}$$

Apparently, this image is in a similar form as the IFM model. Assuming a good linear transformation, both $J_{T}^{k}\left(x\right)$ and $B_{T}^{k}\left(x\right)$ of this image are supposed to be free from color deviations caused by the incident light, i.e., $J_{T}^{k}\left(x\right)$ is close to $J^{k}\left(x\right)$ and $B_{T}^{k}\left(x\right)$ is a constant. So the restoration of $J^{k}\left(x\right)$ can be applied by estimating and eliminating $t^{k}\left(x\right)$ (TM) and $B_{T}^{k}$ (BL) from image $I_{T}^{k}\left(x\right)$. But to ensure a good estimation of $J^{k}\left(x\right)$, priors should be choosen carefully to fit the character of $I_{T}^{k}\left(x\right)$. Moreover, a bad linear transformation is always possible, no matter how robust the transformation method is, so the situation of $J_{T}^{k}\left(x\right)$ being deviated from $J^{k}\left(x\right)$ should also be considered in the restoration process. 

## 3. Proposed Method

**Figure 2:**
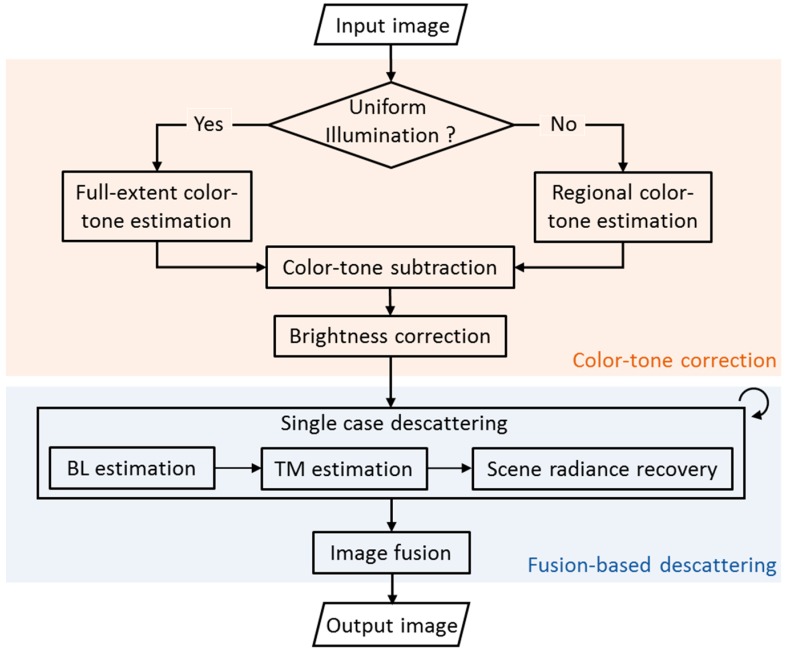
The framework of proposed method.

Based on former analysis, the enhancement of an underwater image with uncertain illumination condition should include two parts: one for suppressing the color deviations originated from the light source, and the other for handling the residual problems for scene radiance estimation. In this section, an enhancement method for underwater images with different illumination conditions is proposed accordingly, with two modules named color-tone correction and fusion-based descattering. The general workflow with key steps in each module is presented in Figure 2. In the following content, details of these modules are introduced.

### 3.1. Color-Tone Correction

#### 3.1.1. General Routine for Uniformly-illuminated Underwater Images

**Figure 3:**
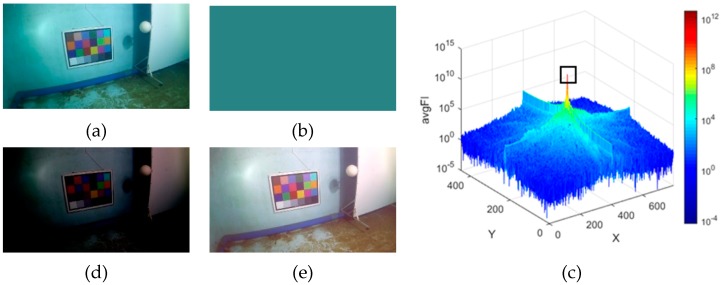
(**a**) A close-up underwater image captured in a water-filled pool. (**b**) Estimated color tone of (**a**). (**c**) The average Fourier frequency of (**a**). The black box in it locates the maximum frequency. (**d**) Color-tone subtracted result of (**a**). (**e**) Brightness-adjusted result of (**d**).

As discussed in the former section, the basis of correcting deviated color tone is to find proper parameters to perform linear transformation of underwater image intensities. Based on our observation, the most frequent color in an equidistant underwater image makes a good estimation of the deviated color tone of this image, and by subtracting it from the original image, an image with achromatic color tone that fits the Gray World assumption can be obtained. To find the most frequent color, we calculate the average Fourier frequency of the input underwater image of all channels and apply inverse Fourier transformation to its maximum value. Details of applying this process is provided in Algorithm 1. In Figure 3a–c, an example of obtaining the deviated color tone from a close-up underwater image captured in our experimental pool is presented.
**Algorithm 1.** Full-extent color tone estimation for an uniformly illuminated image**Input:** Uniformly illuminated image $I^{k}\;,k\in \left\{R,G,B\right\}$. **Output:** Color-tone image $T^{k}$. Calculating the Fourier frequency of the input image: $FI^{k}\leftarrow F\left\{I^{k}\right\}$. F{⋅} represents Fourier transform.Calculating the average frequency of all channels:$\;avgFI\;\leftarrow \;\left(FI^{R}+FI^{G}+FI^{B}\right)/3$.Defining the max-frequency filter: Filt  ←[avgFI==max(avgFI)].Applying the max-frequency filter on the input image: $FT^{k}\;\leftarrow FI^{k}.*Filt$.Calculating the intensity of color tone: $T^{k}\leftarrow F^{-1}\left\{FT^{k}\right\}$, $F^{-1}\left\{\cdot \right\}$ represents inverse Fourier transform.

After calculating the deviated color tone in the full image-range, the color-balanced image is obtained by subtracting the deviated color tone from the original image. Result of this step for the original image in Figure 3a is given in Figure 3d. Apparently, this image is much darker than normal images. Here we use a linear histogram stretching to restore the right intensity range. The basic function of linear histogram stretching [25] is given as follows:

(10) $$I_{out}=\left(I_{in}-a\right)\left(\frac{c-d}{b-a}\right)+d,$$

where $I_{in}$ and $I_{out}$ represent the input and output pixel intensities, respectively, a, b, c and d are the minimum and maximum intensity of the input image and the targeted output image, respectively. In this paper, c and d are set to 255 and 0, and a and b are selected in 0~10% and 90~100% in the whole histogram of the input image depending on the brightness level of the original image. The brightness-adjusted result of the image in Figure 3d is given in Figure 3e. Obviously, the result image has more balanced color tone than the original underwater image. 

**Figure 4:**
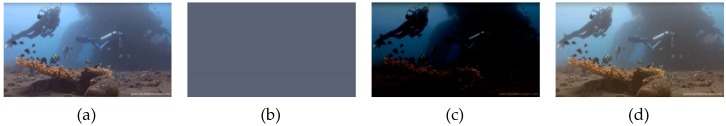
(**a**) The original underwater images from [26]. (**b**) Estimated color tone. (**c**) Color-tone subtracted result. (**d**) Final result of color-tone correction.

For underwater images with uniform illumination and variant scene-camera distance, the above method removes the color deviation induced by the incident light and partly removes the deviation caused by the attenuation along the scene-camera path, as mentioned in the theoretical analysis in former section. In Figure 4, an example of applying the proposed color tone correction method on a uniformly-illuminated and non-equidistant underwater image from Bubble Vision [26] is presented. Compared with the close-up case, the proposed color-tone correction method has managed to remove the global color shift in the input image but left a large amount of regional color deviation and scattering in the processed result. These problems are caused by the variant scene-camera distance, i.e., variant transmission rate of the input image, and will be handled in next module of image descattering.

#### 3.1.2. Regional Color-Tone Estimation for Underwater Images with Non-Uniform Illumination

**Figure 5:**
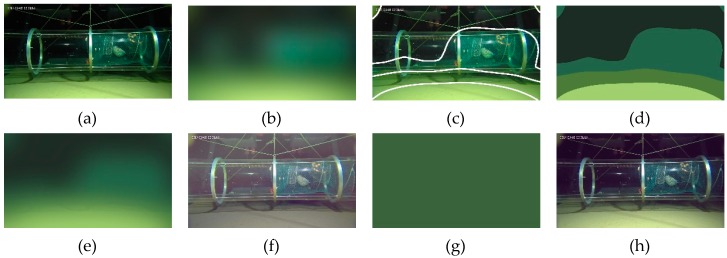
(**a**) An underwater image that was captured in deep sea with only inhomogeneous artificial illumination. (**b**) A raw color tone estimation of (**a**) obtained by applying spatial Gaussian filter. (**c**) Segmenting (**a**) into regions with nearly-uniform illumination by K-means. (**d**) A combination of estimated color tones of all regions. (**e**) Ultimate color-tone image obtained by applying spatial Gaussian filter and guided filter to (d). (**f**) Color-tone corrected result of (**a**) by using the color tone in (**e**). (**g**) Estimated color-tone image from (**a**) by applying color-tone estimation method for uniformly-illuminated images. (**h**) Color-tone corrected result of (**a**) by using the color tone in (**g**).

For underwater images with non-uniform illumination, the key to address the problem of illumination variation is to transform it into the color-tone correction of uniformly-illuminated regions, but to define these regions, the spatial distribution of illumination is supposed to known. To break this cycle, we use a spatial Gaussian filter to produce a blurred version of the input image to approximate the real color-tone deviation, then the regions with nearly-uniform illumination are obtained by applying K-means clustering to the raw color-tone image. To ensure spatial continuity and avoid unwanted details, the used spatial Gaussian filter should be large enough. In this paper, the standard deviation of the Gaussian filter is set to about 1/8 of the shorter-side length of the input image, and the filter size is set to about 4 times the value of standard deviation. The number of clusters is determined intuitively according to the light pattern in the original image, but in most cases, at least three clusters are needed to define a bright region, a dim region, and an intermediate region. After the definition of uniformly illuminated regions, color-tone deviations of these regions are estimated with the same frequency-based method as in the former case. To avoid discontinuities in the ultimate color tone corrected result, the obtained regional color tone image is modified with another spatial Gaussian filter using the same parameters as in the first step to remove noticeable boundaries and with a guided filter [27] to refine the shape of light patterns, in case these patterns are changed in the previous step. A detailed description of this procedure is given in Algorithm 2. In Figure 5a–e, an example of estimating deviated color tone in an underwater image with inhomogeneous artificial illumination is presented with images of key steps of this procedure. In this experiment, the input image was captured in Mariana at the depth of 8200 m during the TS-03 Cruise of the Chinese Academy of Sciences, with illumination completely provided by inhomogeneous and chromatic artificial light sources.
**Algorithm 2**. Regional color-tone estimation for a non-uniformly illuminated image**Input:** Non-uniformly illuminated image $I^{k}\;,k\in \left\{R,G,B\right\}$.
**Output:** Color-tone image $T^{k}$.
Applying a spatial Gaussian filter to $I^{k}$ to get a raw color-tone image $G^{k}$.Segmenting $I^{k}$ into M regions by the K-means method based on the Euclidian distance of colors in $G^{k}$.Initializing $T^{k}$ with an all-zero matrix of the same size as $I^{k}$.For m=1 to M doReshaping the pixels of region m of $I^{k}$ into vector $V_{m}^{k}$.Estimating the color tone $T_{m}^{k}$ from vector $V_{m}^{k}$ by using the frequency-based method in **Algorithm 2**.Replacing the intensity values of pixels in region m of $T^{k}$ with $T_{m}^{k}$.End forApplying a spatial Gaussian filter and a guided filter to remove boundaries and refine $T^{k}$. The guidance image for the guided filtering is the input image $I^{k}$.

After the calculation of deviated color tone, the color-tone corrected result is obtained in the same way as in the former case, i.e., subtracting the estimated color tone from the original image and apply linear histogram stretching to restore a normal brightness level. In Figure 5f, the color-tone corrected result of the original image in Figure 5a is presented. For comparison, the color-tone estimation and correction results obtained via former method for uniformly-illuminated underwater images are also provided in Figure 5g,h. By comparing these two color-tone-corrected results, it is very clear that using the regional color-tone estimation method proposed in this subsection can better restore the color and brightness balance of the non-uniformly illuminated image, and improve the visibility in regions with limited illumination. 

### 3.2. Fusion-Based Descattering

As shown in Figure 4 and Figure 5, a non-equidistant underwater image after being applied with color-tone correction can still present some degradation problems like scattering and regional color deviation, due to the unsettled problems like the variation of transmission rate. According to the theoretical analysis in Section 2, these degradation problems can be solved by image descattering, i.e., estimating TM and BL from input image and recovering scene radiance from it. Mathematically, the descattering of a color-tone-corrected image can be represented with:

(11) $$J_{T}\left(x\right)=\frac{I_{T}\left(x\right)-B_{T}}{t\left(x\right)}+B_{T},$$

where t(x) is the TM, $I_{T}\left(x\right)$, $J_{T}\left(x\right)$ and $B_{T}$ are respectively the linear transformation results of the original image I(x), the deviated scene radiance $L\left(\Omega_{x}\right)J\left(x\right)$ and the deviated BL $B\left(\Omega_{x}\right)$, as demonstrated in Equation (9). 

Essentially, by applying this descattering process, the ultimate goal is to achieve a good estimation of J(x) by recovering $J_{T}\left(x\right)$ from $I_{T}\left(x\right)$. Considering the uncertain states of $J_{T}\left(x\right)$ with respect to the true  J(x), the following three cases are included in the descattering process:

(1) Case of Dim & Scattering images (Case DS): $J_{T}\left(x\right)$ is close to  J(x), and $B_{T}$ is low. In this case, distant regions of $I_{T}\left(x\right)$ are low-contrast and dim, due to the low level of incident and backscattering light. Considering the relativeness between the low contrast and long scene-camera distance, TM and BL can be estimated based on the level of intensity variation of input image [17].
(2) Case of Bright & Scattering images (Case BS): $J_{T}\left(x\right)$ is close to  J(x), and $B_{T}$ is bright. For this case, the high level of ambient light causes high intensity and low contrast in distant regions. Since this case is very close to the outdoor hazy image, the estimation of TM and BL can be done based on the Dark Channel Prior [12].
(3) Case of Color-Cast images (Case CC): $J_{T}\left(x\right)$ is largely biased from  J(x). This is a complement case for images with under- or over-corrected color tone. Since the low-contrast trait is still valid in this case, BL is estimated in the same way as in Case DS. For the calculation of TM, the estimation of scene-camera distance is made in the red channel, because red light is more sensitive to distance change in underwater environment.

**Figure 6:**
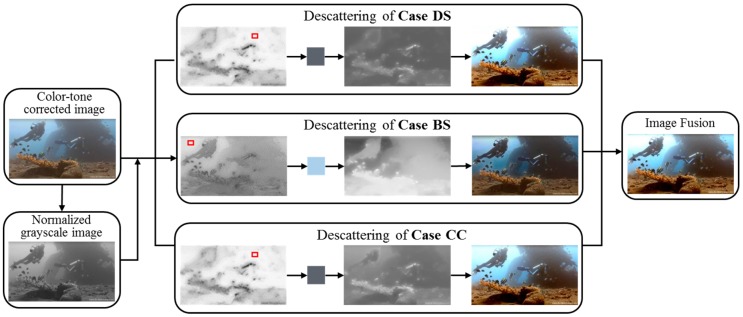
A brief workflow of the fusion-based descattering module.

Since no hard boundaries exist between these three cases, the descattering results of these cases are combined together to form an ultimate enhancement result. The overall workflow of this module is briefly shown in Figure 6. In the following content, details of this workflow are presented.

#### 3.2.1. Descattering of Case DS

As mentioned above, distant regions of this case are dim and low-contrast. Since BL usually corresponds to the farthest region, it can be estimated by finding the region with the lowest intensity variation. The definition of regional intensity variation (RIV) is given as follows, which is modified from the “blurriness” feature defined in [17]:

(12) $$P_{RIV}\left(x\right)=1-N\left\{G\left\{ℋ\left\{\underset{y\in \Omega_{x}}{max}\left(\frac{1}{n}\sum_{i=1}^{n}\left|P_{NG}\left(y\right)-G^{r_{i},r_{i}}\left(y\right)\right|\right)\right\}\right\}\right\}.$$

Here $P_{NG}$ represents the normalized grayscale version of input image. $G^{r_{i},r_{i}}$ is obtained by filtering $P_{NG}$ by an $r_{i}\times r_{i}$ spatial Gaussian filter with variance $r_{i}^{2}$ ($r_{i}=2^{i}n+1$, n is set to 4). This image represents the average intensity level of surrounding region of a given pixel. $\Omega_{x}$ represents a square neighborhood of pixel x, whose size is about 1.5% of the input image. And functions ℋ{⋅}, G{⋅} and N{⋅} are respectively hole-filling operator, guided filtering function and min-max normalization function. By applying this calculation, the level of the regional variation of a pixel is quantified by a 0-1 number with higher values representing lower variations.

The candidates of BL can be found in the region with the highest RIVs. To robustly locate this region, a quad-tree subdivision [17] is applied to $P_{RIV}\left(x\right)$. Based on our experiments, an efficient estimation of BL can be made by setting the ultimate region size to 0.1% of the input image, so the iteration time is usually set to 5. The value of BL is obtained by calculating the average value of pixel intensities in candidate region. Mathematically,

(13) $$B_{T,DS}=mean\left(I_{T}\left(\Omega_{x}\right)\right),\;where\;\Omega_{x}=\underset{x\;\left(QT\right)}{argmax}\left(P_{RIV}\left(x\right)\right).$$

Since $P_{RIV}\left(x\right)$ is a normalized quantification of the level of intensity variation, it can be seen as an approximation of the relative distance between scene and camera, and used for the calculation of TM. The formula of TM estimation is given by:

(14) $$t_{DS}\left(x\right)=e^{-\alpha P_{RIV}\left(x\right)-\beta },$$

where α and β are used to control the variation range of TM. In this paper, their values are set as 0.95 and 0, respectively.

Considering the uneven-attenuation nature of light underwater, the TM of different channels should be set differently. Based on the theoretical analysis in [24], the relationship of TMs of different channels is

(15) $$\frac{ln\left(t_{DS}^{k1}\left(x\right)\right)}{ln\left(t_{DS}^{k2}\left(x\right)\right)}=\frac{B_{T,DS}^{k2}\left(p\lambda^{k1}+q\right)}{B_{T,DS}^{k1}\;\left(p\lambda^{k2}+q\right)},$$

where k1, k2∈{R,G,B} and k1≠k2, p=−0.00113, q=1.62517, $\lambda^{R}=620\;nm,\;\lambda^{G}=540\;nm$ and $\lambda^{B}=450\;nm$. Since the red channel is most sensitive to underwater attenuation, the TM calculated in Equation (14) is assigned to the red channel, and the other two TMs are derived from it based on Equation (15).

The final step of descattering is to recover scene radiance from the given image. The basic formula of this process is shown in Equation (11), but for improving the robustness of scene-radiance recovery, a lower bound of TM is used to avoid dividing extremely small values [12]. The formula of this step is given as follows:

(16) $$\;J_{T,DS}\left(x\right)=\frac{I_{T}\left(x\right)-B_{T,DS}}{max\left(t_{0},t_{DS}\left(x\right)\right)}+B_{T,DS}.$$

where $t_{0}$ is the lower bound of TM and is set to 0.1 in this paper. 

In the first row of Figure 6, an example of applying descattering to Case DS is shown. Following the direction of arrows, the first image is the map of $P_{RIV}\left(x\right)$, the second image shows the color of $B_{T,DS}$, the third image is the $t_{DS}\left(x\right)$, and the last image is the recovered $\;J_{T,DS}\left(x\right)$. The red box in the first image indicates the candidate region of BL estimation. Compared to the input image at the far left of Figure 6, the recovered image is much brighter and has better contrast, especially in the background areas.

#### 3.2.2. Descattering of Case BS

The descattering of Case BS is similar to the dehazing process due to the similarity of Case BS and outdoor hazy images. According to the dehazing method in [12], both BL and TM are estimated from the dark channel of input image to avoid the interference of bright objects in the foreground, but based on former calculation, the estimation of BL can be simplified. As mentioned in the previous subsection, RIV is related to the variation level of pixel intensities within a given region. Since foreground regions are better contrast and richer in details, their RIVs tend to be lower than background regions, so by adding this feature to image intensity, foreground regions can be excluded and pixels with high brightness and long distance can be located. Mathematically, the newly derived feature is given by:

(17) $$P_{N}\left(x\right)=\left[P_{NG}\left(x\right)+P_{RIV}\left(x\right)\right]/2.$$

Candidates of BL are then obtained by finding the top 0.1% pixels in $P_{N}\left(x\right)$, and the value of BL is calculated from the average of candidate intensities, i.e.,

(18) $$B_{T,BS}=mean\left(I_{T}\left(\Omega_{x}\right)\right),\;where\;\Omega_{x}=\underset{x\;\left(0.1\%\right)}{argmax}\left(P_{N}\left(x\right)\right).$$

The estimation of TM is similar to that in DCP-based dehazing method [12], which includes dividing the input image with BL and calculating the Dark Channel from it. The formula of TM calculation is given as follows:

(19) $$t_{BS}\left(x\right)=1-\sigma \underset{k\in \left\{R,G,B\right\}}{min}\left[\underset{y\in \Omega_{x}}{min}\left(I_{T}^{k}\left(y\right)/B_{T,BS}^{k}\right)\right],$$

where σ is used to preserve a small portion of scattering for human eyes to perceive depth, and its value is set as 0.95 in this paper. The size of $\Omega_{x}$ is the same as in the calculation of $P_{RIV}\left(x\right)$. 

Due to the uneven-attenuation of light in water and high sensitivity of red light, the $t_{BS}\left(x\right)$ calculated from Equation (19) is only assigned to the red channel and processed with Equation (15) to derive TMs for the other two channels, as has been done for Case DS. The recovery of scene radiance is also similar, by replacing the BL and TM in Equation (16) with those from this subsection, the scene radiance of this case is obtained. The recovered scene radiance is denoted as $\;J_{T,BS}$. The sample images of this case is shown in the second row of Figure 6 with the same order as in the previous case. As shown by these images, the main function of this case is to reduce the intensities in distant regions and produce a higher-contrast result.

#### 3.2.3. Descattering of Case CC

As mentioned earlier, the estimation of BL in this case is the same as that in Case DS, due to the low contrast of distant regions caused by backscattering, and for the estimation of TM, the red channel is used because it is more sensitive to distance change in underwater environment than the other two channels. 

As the RIV in Case DS, the intensity of red channel is used to produce a rough estimation of the relative distance between the scene and the camera. Since it attenuates severely, we use its regional maximum to indicate the distance of corresponding pixel. Likewise, it is also processed with a guided filter to remove the block artifact and a min-max normalization function to map its value to the range of 0-1. Mathematically, the relative distance calculated from the red channel is defined as:

(20) $$d_{R}\left(x\right)=1-N\left\{G\left\{\underset{y\in \Omega \left(x\right)}{max}\left(I_{T}^{R}\left(y\right)\right)\right\}\right\}.$$

The size of $\Omega_{x}$ here is still the same as that for calculating $P_{RIV}\left(x\right)$.

With the relative distance calculated, TM of this case is obtained by replacing the $P_{RIV}\left(x\right)$ in Equation (14). The following process is identical with former two cases, in which the calculated TM is assigned to the red channel and used to derive the values for the other two channels, and the scene radiance is recovered by replacing corresponding parameters in Equation (16). The recovered scene radiance is denoted as $\;J_{T,CC}$.

Images of this case is shown in the last row of Figure 6. Since the BL of this case is the same as that in Case DS, the resulted images of these two cases has similar color tones. But due to the difference of TM estimation, the descattering of this case emphasizes more on color correction than contrast improvement, which leads to a plainer result than Case DS.

#### 3.2.4. Image Fusion

Since there is no hard boundaries between the previous three cases, all of the descattered images are fused together to form a final enhancement result. Inspired by [17], these images are fused linearly with weights calculated from two sigmoid functions which respectively describe the level of brightness and color balance of the input image. The formula of image fusion is given as follows:

(21) $$J_{fusion}=\theta_{1}\cdot J_{T,BS}+\left(1-\theta_{1}\right)\cdot \left[\theta_{2}\cdot J_{T,CC}+\left(1-\theta_{2}\right)\cdot J_{T,DS}\;\right],$$

where $\theta_{1}$ is a sigmoid function related to the general brightness level of input image:

(22) $$\theta_{1}={\left\{1+exp\left[-\omega \left(avg\left(I_{T}\right)-\tau I_{M}\right)\right]\right\}}^{-1},$$

with ω controls the flatness of the sigmoid function and τ controls the base level of image brightness. In this paper, their values are set as 20 and 0.6, respectively. $I_{M}$ represents the maximum gray scale level, it equals to 255 for images with 8-bit pixels. The second weight $\theta_{2}$ is about the level of color bias in the input image. It is defined as follows:

(23) $$\theta_{2}={\left\{1+exp\left[-\omega \left(\underset{k\in \left\{R,G,B\right\}}{max}avg\left(I_{T}^{k}\right)-\underset{k\in \left\{R,G,B\right\}}{min}avg\left(I_{T}^{k}\right)-\gamma \cdot avg\left(I_{T}\right)\right)\right]\right\}}^{-1},$$

where γ controls the basic metric of color bias, and is set as 0.1 in this paper.

The sample image of this step is shown at the far right of Figure 6. As indicated by the arrows, it is the combination of descattered results of Case DS, Case BS and Case CC. In this fusion, the calculated weights for the three cases are respectively 0.4529, 0.2141 and 0.333, so the result image is more similar to the output of Case DS and Case CC, where the low contrast, low intensity and color deviation problems are tackled. 

## 4. Evaluation and Results

In this section, the proposed method is experimented on three different datasets, including a shallow water dataset from Bubble Vision (BV) [26], a laboratory dataset from a deep-sea experiment pool (EP), and a real deep sea (DS) dataset from the TS-03 Cruise of the Chinese Academy of Sciences. Among these datasets, the BV dataset was captured in Bali under uniform and natural light, the EP dataset was captured in a 22 m×10 m×20 m water-filled pool at night, and the DS dataset was captured in open water of Mariana at the depth over 8200m. For the latter two datasets, illumination was provided by artificial lighting devices located on the photographed objects or near the camera, and light bulbs of green or blue colors were used to extend vision range while saving energy. These illumination settings caused additional color deviation and light imbalance problems to images of these datasets. 

The performance of proposed method is evaluated qualitatively and quantitatively in terms of visual quality and practical value, which include the assessment of color richness, color balance and contrast of enhanced images, and their credibility and information content, especially in badly-illuminated regions. The performance of proposed method is compared with the fusion-based method from Ancuti et al. [9], the Red-Channel method from Galdran et al. [13] and the IFM-based method of Peng and Cosman [17]. These methods are selected for their reported good performance on underwater image enhancement. Specifically, Galdran’s method is able to enhance normal underwater images as well as those with artificial light sources, and Peng’s method is able to enhance underwater images with various lighting conditions. In the following content, the evaluation results of these methods on underwater images with different illumination conditions are reported.

### 4.1. Evaluation on the Dataset of Uniform Natural Illumination

**Figure 7:**
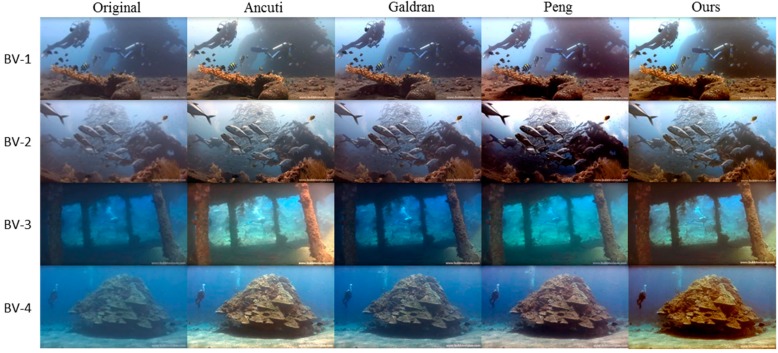
Visual comparison of different methods on enhancing uniformly-illuminated underwater images from the BV dataset.

The evaluation on uniformly-illuminated underwater images is conducted on the BV dataset. In Figure 7, enhancement results of Ancuti’s method, Galdran’s method, Peng’s method and the proposed method are presented, together with the original images from the BV datasets. As shown in this figure, Ancuti’s method removes the bluish hue and broads the range of color, but the low contrast problem of distant regions is not solved. Galdran’s method, on the contrast, improves the image contrast but cannot fully remove the bluish hue. Peng’s method achieves improvements in both aspects, but it can be unstable and cause over-enhanced results, such as that of BV-2. In comparison, our method is robust and can improve both color balance and image contrast. Moreover, our method is also better at preserving details during the enhancement, such as the fish school in BV-2 and the bubble group in BV-3.

Since visual evaluation can be influenced by the size of image and the color temperature of the displayer, we also use a quantitative metric to objectively evaluate the image quality. The selected metric is the UIQM from [28], which is a non-reference measure of the image visual quality based on evaluations of color balance and richness, detail sharpness and image contrast. The formula of UIQM is given by:

(24) $$UIQM=c_{1}\times UICM+c_{2}\times UISM+c_{3}\times UIConM,$$

where UICM, UISM and UIConM are respectively the measures of image colorfulness, image sharpness and image contrast. The weights of these measures are assigned as $c_{1}=0.0282$, $c_{2}=0.2953$ and $c_{3}=3.5753$, according to [28]. The UIQM is calculated for every image in this experiment, but to avoid an overlong table, the scores of individual images are listed in Appendix A (Table A1), and in the main text, only the average scores of each method are presented (Table 1). Apparently, the quantitative evaluation results are in accordance with previous qualitative evaluation. As shown in Table 1, the highest scores of UICM, UIConM and UIQM are won by our results, which means our method outperforms the others in terms of color richness and balance, contrast, and the overall visual quality of image. In the evaluation of sharpness, our method has the highest values in BV-3 and BV-4, but is behind Peng’s method in BV-1 and BV-2. This can be caused by the strong descattering of Peng’s results in these two trials.

### 4.2. Evaluation on the Datasets of Non-Uniform Artifial Illumination

The evaluation on underwater images with inhomogeneous illumination is conducted on the EP dataset and the DS dataset. As mentioned above, due to the use of inhomogeneous and chromatic lighting devices, images of these two datasets are more likely to suffer from regional color deviation and lighting imbalance than images in the BV dataset. So in this evaluation, we will not only focus on the quality improvement of the whole image, but also assess the enhancement in severely degraded regions. 

#### 4.2.1. Experiment on the EP Dataset

**Figure 8:**
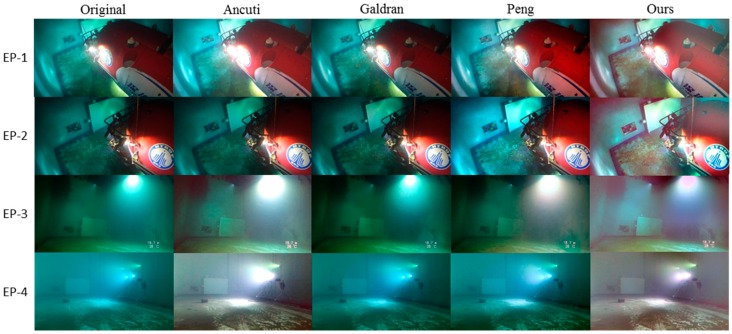
Visual comparison of different methods on enhancing non-uniformly-illuminated underwater images from the EP dataset.

The original EP images and corresponding enhancement results of Ancuti’s method, Galdran’s method, Peng’s method and the proposed method are shown in Figure 8. Clearly, Galdran’s method and Peng’s method fail to restore the right color in this test. Ancuti’s method is also failed in the first two trials. By contrast, our method improves the color balance in all tested images. As can be seen in Figure 8, the blue-green color cast in the original images is largely reduced in our results. For EP-3 and EP-4, a very realistic color tone is restored in our results, and for EP-1 and EP-2, the strong color deviations are largely suppressed, but due to the rapid color change in these images, the result images are less realistic than those of EP-3 and EP-4. Actually, this is a deficiency of the regional color-tone estimation method in our work, which will be solved in future studies hopefully. Apart from color correction, the illumination balance is also improved in our results. For EP-1, EP-2 and EP-3, the low-illumination regions are brighter than before, and for EP-4, the hazy region is of higher contrast. This improvement is a side-product of the regional color-tone correction method in our work. For practical use, this trait can be very helpful since it improves the visibility of badly-illuminated regions without consuming more energy for illumination.

For objective evaluation, UIQM is used to assess the visual quality of enhanced images. The average scores of each method are shown in Table 2, and the scores of individual images are listed in Table A2 of Appendix A. Unlike former experiment, the evaluation scores do not support our visual assessment. Based on the scores, Galdran’s results have the best visual quality and the highest contrast, and Peng’s method has the best performance in terms of colorfulness and sharpness. From the calculation formulas of UICM, UISM and UIConM in [28], we speculate that this disagreement may be caused by the omission of special information in the calculation of these measures. Due to this omission, the regional color deviation and uneven brightness in Galdran’s and Peng’s results are mistaken as high colorfulness and contrast, which leads to high scores in the evaluation results. Unfortunately, no other metric is found to replace UIQM for this case, since the study of regional-deviated underwater image is very rare. 

**Figure 9:**
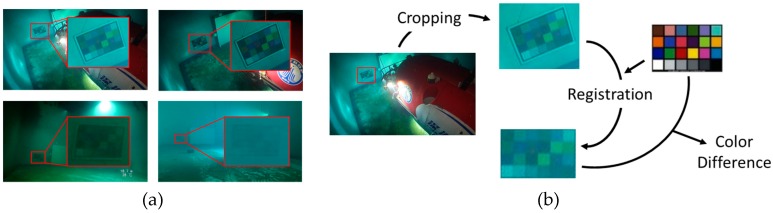
(**a**) The locations of color boards in EP images. (**b**) The procedure of preparing the color board region for calculating the color difference against the ground truth image.

Although the evaluation of overall image quality is unachievable at present, the evaluation of regional color balance can still be made, owing to the color board captured in each EP image. As shown in Figure 9a, the color board locates at the region with uniform and severe color deviation in each image, so by calculating the color difference between the color board and a ground truth image, the degree of color balance of this region can be known. Before calculation, the procedure in Figure 9b is applied to correct the affine deformation of the color board in each image. The metric used to evaluate color difference is the mean square error (MSE), which is given by:

(25) $$MSE=\frac{1}{N}\overset{N}{\underset{x=1}{\sum }}{\left(I\left(x\right)-I_{0}\left(x\right)\right)}^{2},$$

where $I_{0}\left(x\right)$ and I(x) are respectively the pixel values of the ground truth image and the image to evaluate. The average and individual MSEs for each method are listed in Table 2 and Table A2 (Appendix A), respective. As shown by these values, the color boards of our results have the lowest MSEs. Since the color tones of color board regions are nearly uniform, this evaluation result shows that our method can restore the regional color balance better than the other methods. This result is consistent with former visual observation.

#### 4.2.2. Experiment on the DS Dataset

**Figure 10:**
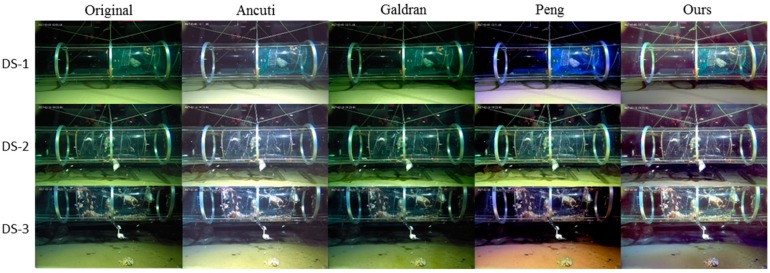
Visual comparison of different methods on enhancing non-uniformly-illuminated underwater images from the DS dataset.

The original DS images and corresponding enhancement results of the four tested methods are shown in Figure 10. Due to the lack of ambient light, the DS images present less scattering but stronger brightness imbalance than the EP images. Similar to the former case, Galdran’s method and Peng’s method fail to enhance the original images in terms of the color balance and the brightness balance. Ancuti’s method removes the color cast in the foreground but only slightly improves the illumination in the background. Our method restores the color balance as well as the illumination balance. As can be easily noticed, in our results, the greenish tone in the foreground is completely removed, and the colors and shapes in the background are much more salient than before. 

To keep the consistency of the evaluation procedure, the UIQM measures are still calculated for images in this experiment. The average and individual scores of this evaluation are shown in Table 3 and Table A3 (Appendix A), respectively. Due to the large span of color and strong brightness imbalance, the highest scores of this evaluation are won by Peng’s results.

To evaluate the enhancement of dark regions in the original images, we calculate the entropy for all images in this experiment. In information theory, entropy measures the “randomness” of a system. And for image enhancement tasks, the increase of image entropy indicates larger information content and higher distinguishability of details in the enhanced images [29,30]. The entropy of an 8-bit gray-scale image is defined as follows:

(26) $$entropy=-\overset{255}{\underset{i=0}{\sum }}p_{i}{log}_{2}p_{i}.$$

Here $p_{i}$ is the probability of the gray level i in a pixel of the image, which is calculated by:

(27) $$p_{i}=\frac{\sum_{x=1}^{N}\left(I\left(x\right)==i\right)}{\sum_{j=0}^{255}\sum_{x=1}^{N}\left(I\left(x\right)==j\right)},$$

where I(x) represents the pixel value of the image and N is the total number of pixels in the image. To measure the entropy of an RGB image, we first calculate the entropies of the three color channels separately, then calculate the average of them as the entropy of the whole image.

The average and individual entropies of images in the experiment are listed in Table 3 and Table A3 (Appendix A), respectively. As shown in these tables, our method improves the image entropies better than the other methods. This result is consistent with former visual evaluation that our method can effectively improve the information content in low-brightness regions. Ancuti’s results come the second in this evaluation, which is due to the less salient backgrounds in these images.

## 5. Conclusions

In this paper, we proposed an enhancement method for underwater images with uniform or non-uniform illumination conditions. In practical operations, these conditions usually correspond to the shallow-water environment and the deep-sea environment, respectively. The proposed method is composed of two modules: color-tone correction and fusion-based descattering. The first module reduces the regional or full-extent color-tone deviation that is caused by different types of incident light. And the second module solves the problems of low contrast and pixel-wise color deviation that are left after applying the first module. The proposed method is experimented on laboratory and open-water images under different depths and illumination states. Qualitative and quantitative evaluations show that the proposed method outperforms many other methods in improving the color balance and contrast of underwater images with different illumination conditions, and is especially effective in improving the color accuracy and information content in badly-illuminated regions of underwater images with non-uniform illumination, which are commonly seen in deep-sea researches and operations.

## Figures and Tables

**Table 1:** Quantitative evaluation on the BV dataset in terms of UICM, UISM, UIConM and UIQM.

	Original	Ancuti	Galdran	Peng	Ours
UICM	3.2343	5.5697	4.2195	5.1658	7.0555
UISM	0.0040	0.1535	0.0099	0.5475	0.2874
UIConM	0.1518	0.1602	0.1913	0.8654	2.5721
UIQM	0.6352	0.7752	0.8060	3.4016	9.4797

**Table 2:** Quantitative evaluation on the EP dataset in terms of general visual quality (UICM, UISM, UIConM and UIQM) and regional color accuracy (MSE).

	Original	Ancuti	Galdran	Peng	Ours
UICM	5.0334	6.3860	5.3108	7.2688	5.5161
UISM	0.1178	0.0010	0.1066	0.3310	0.0871
UIConM	4.2431	0.8215	7.0206	5.5843	2.0639
UIQM	15.3471	3.1176	25.2820	20.2681	7.5602
MSE	7432	5193	7784	6604	3339

**Table 3:** Quantitative evaluation on the DS dataset in terms of visual quality (UICM, UISM, UIConM and UIQM) and image entropy.

	Original	Ancuti	Galdran	Peng	Ours
UICM	4.2310	4.7390	4.1804	6.2793	3.4511
UISM	0.1869	0.0020	0.3570	0.4239	0.0798
UIConM	0.6012	0.1710	0.5157	1.2234	0.6491
UIQM	2.3240	0.7456	2.0669	4.6467	2.4415
Entropy	6.9938	7.3031	7.0108	6.9897	7.4274

## Appendix A. Quantitative Evaluation Scores of Individual Images in Section 4

**Table A1:** UICM, UISM, UIConM and UIQM scores of original BV images and corresponding enhanced images of tested methods.

		Original	Ancuti	Galdran	Peng	Ours
UICM	BV-1	4.2379	4.8382	4.7165	5.6624	7.2188
	BV-2	3.2439	3.2660	3.6066	3.9531	5.5196
	BV-3	3.2474	6.9919	4.4179	5.4154	7.9651
	BV-4	2.2081	7.1829	4.1371	5.6324	7.5185
UISM	BV-1	0.0051	0.1469	0.0127	1.3273	0.4114
	BV-2	0.0051	0.4581	0.0132	0.8543	0.4969
	BV-3	0.0039	0.0053	0.0066	0.0048	0.0404
	BV-4	0.0020	0.0038	0.0071	0.0038	0.2010
UIConM	BV-1	0.1518	0.1518	0.1518	1.7274	2.2891
	BV-2	0.1518	0.1854	0.3099	1.1367	3.3554
	BV-3	0.1521	0.1521	0.1521	0.4462	2.8458
	BV-4	0.1515	0.1515	0.1515	0.1515	1.7980
UIQM	BV-1	0.6639	0.7227	0.6796	6.7275	8.5091
	BV-2	0.6358	0.8904	1.2136	4.4277	12.2988
	BV-3	0.6364	0.7424	0.6703	1.7493	10.4113
	BV-4	0.6046	0.7454	0.6605	0.7017	6.6997

**Table A2:** UICM, UISM, UIConM, UIQM and color board MSE scores of original EP images and corresponding enhanced images of tested methods.

		Original	Ancuti	Galdran	Peng	Ours
UICM	EP-1	8.4246	10.7532	8.0766	11.9112	6.5124
	EP-2	8.0984	8.6927	8.0727	12.6849	8.2544
	EP-3	2.4301	3.1563	2.8046	2.4072	4.7343
	EP-4	1.1803	2.9416	2.2895	2.0720	2.5635
UISM	EP-1	0.0007	0.0003	0.0007	0.9312	0.0005
	EP-2	0.2219	0.0004	0.2220	0.2045	0.3437
	EP-3	0.0033	0.0020	0.0033	0.0028	0.0024
	EP-4	0.2450	0.0014	0.2002	0.1854	0.0017
UIConM	EP-1	0.8004	0.7899	0.8574	5.2734	0.7899
	EP-2	1.8839	0.7899	4.9738	3.6223	2.2100
	EP-3	4.6511	0.7899	9.2699	4.1454	4.3392
	EP-4	9.6370	0.9164	12.9814	9.2959	0.9164
UIQM	EP-1	3.0993	3.1275	3.2934	19.4650	3.0079
	EP-2	7.0295	3.0694	18.0759	13.3689	8.2357
	EP-3	16.6985	2.9137	33.2227	14.8899	15.6480
	EP-4	34.5609	3.3597	46.5362	33.3487	3.3491
MSE	EP-1	4957	6228	4046	3890	2454
	EP-2	6413	3901	6642	4117	2039
	EP-3	10158	5740	11138	9222	4172
	EP-4	8201	4902	9310	9186	4690

**Table A3:** UICM, UISM, UIConM, and UIQM scores and entropies of original DS images and corresponding enhanced images of tested methods.

		Original	Ancuti	Galdran	Peng	Ours
UICM	DS-1	4.4438	4.1235	4.3040	6.5791	3.4382
	DS-2	3.8316	4.0959	3.5961	5.5181	3.9264
	DS-3	4.4177	5.9976	4.6412	6.7406	2.9886
UISM	DS-1	0.5532	0.0023	0.2578	0.3726	0.1347
	DS-2	0.0034	0.0017	0.3608	0.4498	0.0967
	DS-3	0.0042	0.0020	0.4522	0.4494	0.0080
UIConM	DS-1	1.5033	0.2127	0.9931	2.2727	1.2113
	DS-2	0.1502	0.1502	0.3078	0.9686	0.5435
	DS-3	0.1502	0.1502	0.2461	0.4290	0.1924
UIQM	DS-1	5.6635	0.8773	3.7481	8.4210	4.4674
	DS-2	0.6459	0.6529	1.3084	3.7516	2.0824
	DS-3	0.6627	0.7066	1.1443	1.8566	0.7746
Entropy	DS-1	6.9979	7.2007	7.0056	6.5553	7.3432
	DS-2	6.9299	7.2192	6.9439	7.5355	7.4390
	DS-3	7.0536	7.4894	7.0830	6.8783	7.5001
